# Presentation and Management of a Complex Orbital Apex Syndrome From Penetrating Mechanical Spring Hook: A Case Report

**DOI:** 10.7759/cureus.29630

**Published:** 2022-09-26

**Authors:** Iyad Majid, Melanie Martel

**Affiliations:** 1 Biochemistry, University of California Santa Barbara, Granite Bay, USA; 2 Ophthalmology, University of California Davis School of Medicine, Sacramento, USA

**Keywords:** lamina papyracea, superior orbital fissure syndrome, orbital apex syndrome, intraorbital foreign body, penetrating orbital injury

## Abstract

Our case report demonstrates the management of a unique penetrating orbital injury. The intraorbital foreign body was an approximately 22 cm long metal dishwasher spring hook lodged into the left orbital apex. An ophthalmological check-up a couple of weeks following the removal surgery discerned the patient had an unprecedented case of orbital apex syndrome. We present this unique case so physicians, medical students, and other emergency and medical professionals can learn about the diagnostic, surgical, and multidisciplinary management necessary to achieve a favorable clinical outcome.

## Introduction

Penetrating orbital injuries (POIs) are uncommon and represent a small portion of the head and eye trauma [1]. Penetrating intraocular foreign bodies (IOFB) may damage orbital and intracranial structures, including “the internal carotid artery (ICA), basilar artery, cavernous sinus, pituitary gland, and cranial nerves I-VI, in addition to the frontal lobe, temporal lobe, and the brain stem,” among other vital organs and bodily features [2]. Prompt assessment, proper surgical care, and immediate ophthalmic evaluation and management are urgent necessities.

Orbital apex syndrome (OAS) is defined as “vision loss from optic neuropathy and ophthalmoplegia due to the involvement of ocular motor nerves in the anatomical region of the orbital apex.” The condition is most typically caused due to an “abnormal autoimmune response,” or bacterial, parasitic, fungal, or viral infection [3]; rarely do ophthalmologists come across a patient with OAS secondary to a POI.

We present a unique, complex case of OAS caused by a high-velocity penetrating injury with a hook-tipped object, where an early multidisciplinary surgical operation was warranted: The IOFB impaled the orbital apex, damaged the optic nerve, and pierced adjacent intracranial tissue.

## Case presentation

A 66-year-old female was brought into the emergency room as a tier 1 trauma alert after being impaled through the left orbital with a 21.5 x 1.7 cm cylindrical gray metal coiled spring with hook-shaped ends. She was fixing an old dishwasher with her friend when a wire was cut, and the foreign body impaled her in her face slightly below the eye. The patient was immediately started on intravenous antibiotics clindamycin and Levaquin, probiotics, and given a tetanus shot.

**Figure 1:**
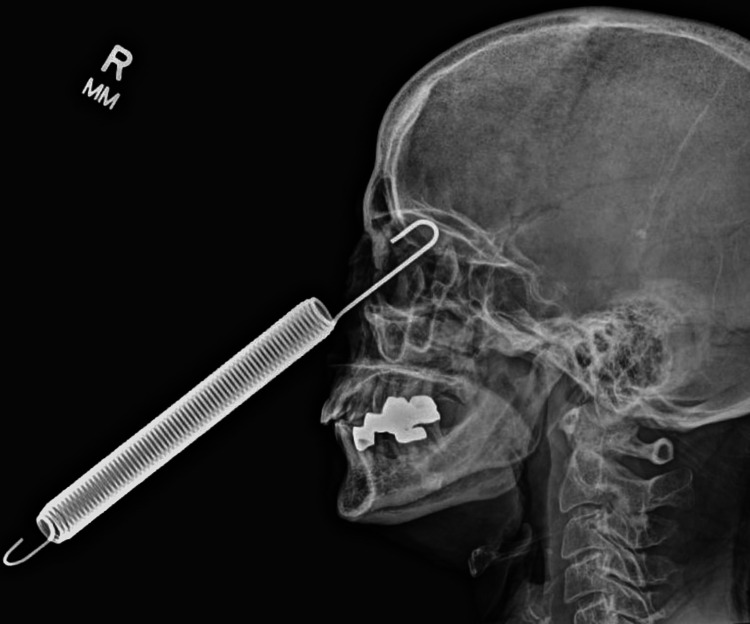
Spring embedded in the left orbit at the base of the skull.

Initial examination revealed the patient had no light perception vision in her left eye. The nature of the injury precluded CT analysis due to the metal artifact. The x-ray of the facial bones revealed the foreign body to have entered the left orbital and extended into the left frontal lobe through the orbital apex (Figure 1). There was a high probability of an imminent or life-threatening deterioration in the patient's condition - due to intracranial involvement - without immediate intervention. Hence, she was immediately taken to the operating room for further evaluation.

The examination under anesthesia revealed the patient’s left pupil was fixed and dilated (to 6 mm) with no sensitivity to light nor any apparent extraocular movements. There was 2 mm of relative left globe proptosis, restricted lateral globe rotation upon forced ductions, and the left global position experienced right exotropia. The examination also revealed commotio retinae to the fundus of the left eye and secondary optic nerve injury. A left globe exploration and orbital decompression surgery were performed. The hook was finally able to be rotated and removed. The wound was then copiously irrigated, and further hemostasis was achieved with bipolar cautery.

**Figure 2:**
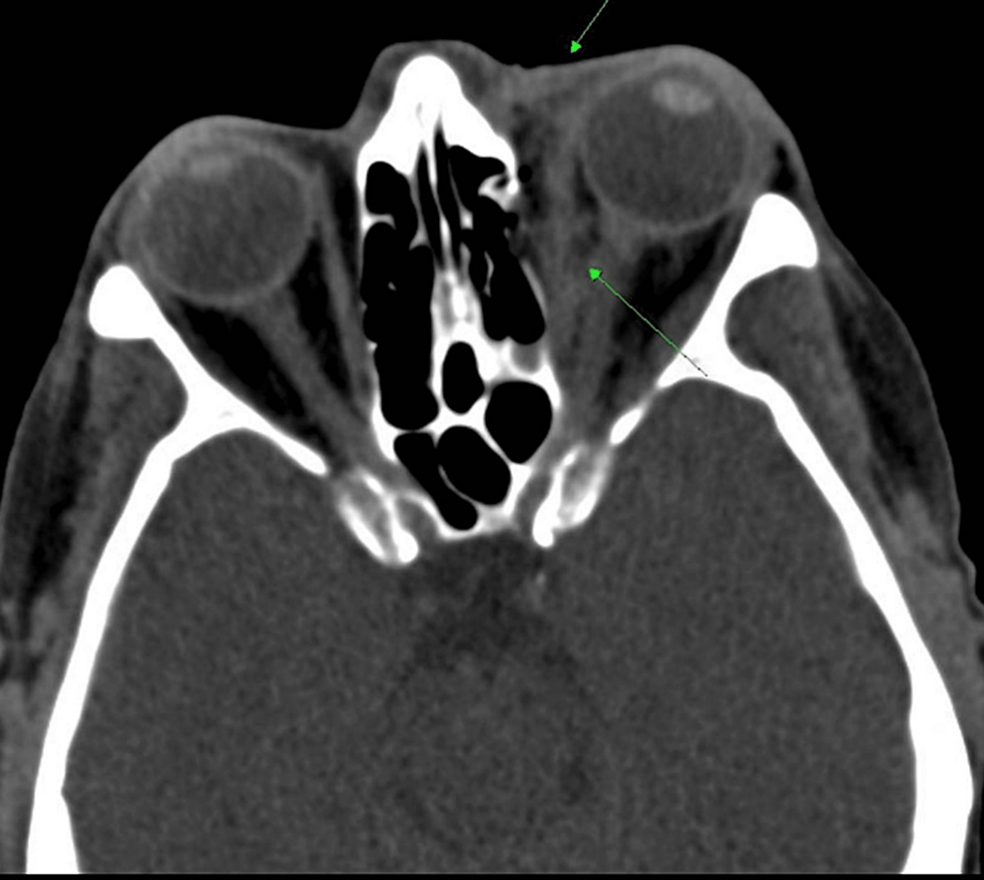
CT of the maxillofacial region reveals stranding of intraorbital fat and periorbital soft tissue swelling of left orbit.

Postoperatively, the patient continued to take her prescribed antibiotics (clindamycin) for five days. Additionally, she was prescribed Fioricet and Tobramycin ointment. CT scans of the maxillofacial region and the head were soon performed with coronal and sagittal reformats (Figure 2). The scan demonstrated a small fracture to the anterior aspect of the left lamina papyracea was discovered. The CT also revealed a small region (10 x 5 mm) of soft tissue density in the medial aspect of the left orbit reflecting a small and soft tissue hematoma adjacent to the orbital wall fracture. Small amounts of fluid and ethmoid air cells caused by the medial orbital wall fracture were found in the ipsilateral paranasal sinuses. There was also mucosal thickening and fluid in the left maxillary sinus. The patient was then discharged the day after her operation.

A couple of weeks following the surgery, the patient was seen for an ophthalmological check-up, where she was clinically diagnosed with OAS (as discussed more thoroughly below).

## Discussion

To our knowledge, we are the first to report a POI case caused by a dishwasher injury. This case not only represents a rare POI by a unique IOFB, but the precise location the hook entered and drove into is also unprecedented. The hook penetrated through the medial rectus muscle, clipped through the orbital apex, and lodged into a really exact, pin-point location: if it entered any further, it would have penetrated the pituitary gland, and any deviation to the right and left would break through the lamina papyracea and completely clip the optic nerve, respectively. Hence, multidisciplinary management of the case was naturally challenging.

Dangers of surgery included ongoing inflammation and infection, and the possibility of losing the left eye. The x-ray of the facial bones before the surgical operation revealed little information regarding the damage caused by the hook and the necessary details for removal. A CT scan would also render unhelpful with the metal foreign body in the orbital, leaving little information on the anatomical neuropathy and orbital manifestation of injury. Thus, the first decision was to immediately cut the protruding segment. However, after further inspection by the ophthalmologist, the best method appeared to be removing the foreign body completely intact to ensure no foreign bodies remain in the orbital.

Second, the tier 1 trauma status of the case gave doctors little time to ask if the patient was allergic to penicillin; thus, proper selection of antibiotics was also crucial. To prevent possible infections from spreading into the brain, the patient received both clindamycin and Levaquin. Clindamycin efficiently combats gram-positive and anaerobic bacteria [3], and Levaquin eliminates remaining gram-negative bacteria types [4]. Finally, a tetanus shot was quickly administered upon arrival at the emergency room.

**Figure 3:**
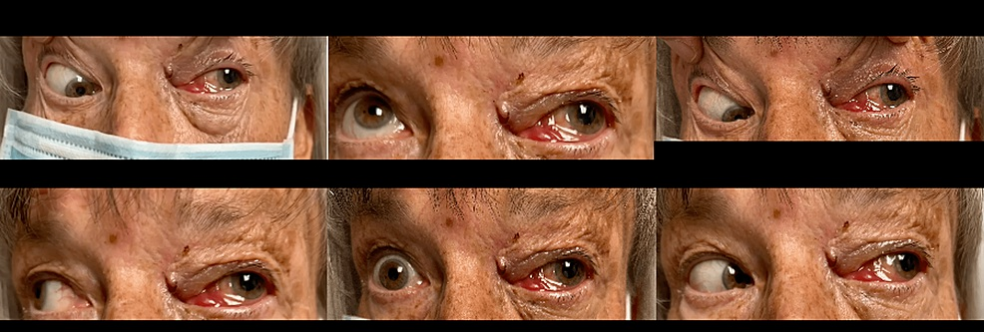
Certain cardinal positions of gaze showing orbital apex syndrome in the left eye.

**Figure 4:**
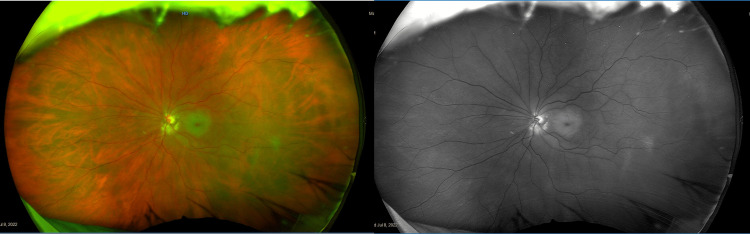
Swelling seen behind the macula with a dark spot hovering over the central artery due to occlusion.

An ophthalmological check-up two weeks post-operation revealed the patient had an unusual case of OAS. Common causes of OAS include fungal, bacterial, viral, or parasitic infections, or autoimmune disorders [5]. Traumatic factors are rare perpetrators [6], and the symptoms that ensue are often mistaken with superior orbital fissure syndrome. While both superior orbital fissure syndrome and OAS involve cranial nerves III, IV, V subdivisions 1, and VI, the main differentiating factor is optic neuropathy in OAS [7]. The patient developed OAS with vision loss from the damage caused by the metal spring hook. The foreign body penetrated through the medial rectus muscle, clipping extraocular movement, and damaging the optic nerve. Photographs taken of the patient in certain cardinal positions of gaze demonstrated the extent of the POI to her extraocular muscle function (Figure 3). Images of the patient’s left retina also produced at the check-up demonstrated a central artery occlusion, most probably responsible for depressed ocular movement and no light perception (Figure 4).

## Conclusions

Both POIs and OAS are individually rare and often uniquely stem from separate factors. However, we report a case that witnesses a combination of the two: a dishwasher spring hook catapulted into the left orbital clipping extraocular movement, damaging the optic nerve, and cutting light perception. Multidisciplinary management was crucial to achieving a favorable clinical outcome.
